# Preventing Deadly Complications of Cardiac Sarcoidosis

**DOI:** 10.7759/cureus.41175

**Published:** 2023-06-30

**Authors:** Gurinder K Sunner, Jude Elsaygh, Kunal Mehta

**Affiliations:** 1 Internal Medicine, New York Presbyterian Brooklyn Methodist Hospital, New York City, USA; 2 Cardiology, New York Presbyterian Brooklyn Methodist Hospital, New York City, USA

**Keywords:** ventricular tachycardia, electrophysiology studies, conduction abnormalities, icd therapy, atrioventricular blocks, cardiac sarcoidosis

## Abstract

Cardiac sarcoidosis is a rare complication of pulmonary sarcoidosis, causing granulomatous inflammation of the myocardium, ultimately resulting in infiltrative cardiomyopathy. Through fibrotic inflammation, cardiac sarcoidosis predisposes patients to multiple complications, such as conduction abnormalities, ventricular arrhythmias, heart failure, and sudden cardiac death. Due to the adverse prognosis of conduction abnormalities in these patients, dual chamber implantable cardiac defibrillator (ICD) therapy is beneficial in reducing mortality and morbidity. Early ICD placement can prevent further immune-driven fibrotic conduction complications. Here we present a case of a 50-year-old female who underwent ICD placement due to conduction abnormalities secondary to cardiac sarcoidosis.

## Introduction

Cardiac sarcoidosis (CS) is a rare manifestation of the many heterogeneous multisystem findings in sarcoidosis. It involves focal areas of inflammation in the myocardium, causing diffuse fibrosis through augmentation by interleukin (IL)-4, IL-10, and transforming growth factor beta (TGF-β). Cardiac involvement in sarcoidosis patients poses a higher risk of mortality as compared to pulmonary complications. Here we present a rare case of significant cardiac complications in a patient with pulmonary sarcoidosis.

## Case presentation

**Figure 1:**
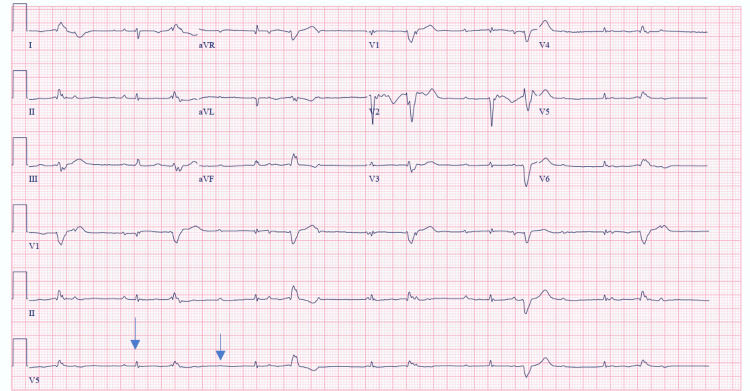
Electrocardiogram with evidence of third degree atrioventricular block

A 50-year-old female with a history of sarcoidosis presented to the emergency department for palpitations and lower extremity swelling. Electrocardiogram showed a third-degree atrioventricular block and low voltage (Figure 1). Further, a transthoracic echocardiogram showed evidence of a large pericardial effusion with severe right ventricular dilation and dysfunction. There was moderate tricuspid regurgitation with an estimated pulmonary arterial systolic pressure of 80mmHg. She underwent a pericardial window and intravenous diuresis with the improvement of her symptoms. An electrophysiology study induced ventricular tachycardia, and she subsequently underwent placement of a dual chamber implantable cardiac defibrillator. Additionally, an endomyocardial biopsy showed evidence of non-caseating granulomas, confirming cardiac sarcoidosis. The patient was eventually discharged on oral steroids and mycophenolate with significant improvement of her functional status outpatient.

## Discussion

Sarcoidosis is an inflammatory disease characterized by non-caseating granulomas, triggering cardiac involvement in up to 25% of cases. Cardiac sarcoidosis is associated with a worse prognosis, and diagnosis can be difficult, requiring a multimodal approach. The condition carries a high incidence of morbidity and mortality due to an increased risk of sudden cardiac death (SCD), which can be caused by conductional abnormalities such as atrioventricular blocks or fatal arrhythmias. The pathological process of CS often leads to fibrosis and inflammation of the myocardium, leading to conduction abnormalities such as bundle branch blocks, atrioventricular (AV) blocks, or ventricular tachycardia (VT). These patients may present with several nonspecific symptoms such as palpitations, syncope, and fatigue, and even though tissue diagnosis may be confirmatory and serves as a poor prognostic factor, endomyocardial biopsy has low sensitivity due to the patchy nature of myocardial granulomas [1].

As a result, advanced cardiac imaging can be used in the initial evaluation as a non-invasive detection modality. Initial evaluation can begin with transthoracic echocardiography with evidence of a restrictive filling pattern or diastolic dysfunction, even though it has a low sensitivity and specificity [1]. Of note, the most important clinical predictor of mortality in these patients is left ventricular ejection fraction [2]. Cardiac magnetic resonance imaging (cMRI) is more useful due to its high sensitivity, however. Late gadolinium enhancement, indicative of fibrosis, and contiguous extension from the left to right ventricle can increase the sensitivity of cardiac MRI. Further, positron emission tomography (PET) can complement diagnosis and further predict prognosis. PET findings concerning for cardiac sarcoidosis are associated with an increased risk of ventricular arrhythmias and sudden cardiac death. Serial PET can also be useful in monitoring response to treatment. Lastly, electrophysiology studies play a vital role in diagnosis but further aid in risk stratification and management of arrhythmias or conduction disturbances [1]. Due to their ability to identify the location and extent of cardiac involvement, they can help identify patients who are eligible for implantable cardioverter defibrillator (ICD) therapy, cardiac resynchronization therapy (CRT), or medical therapy, ultimately reducing sudden cardiac death [3].

Management of CS is usually centered around immunosuppression with corticosteroids; however, due to the long-term side effects associated with corticosteroid therapy, steroid-sparing therapies can be considered for adjunctive therapy. Even though there has not been extensive data on which agents may be the most beneficial, several immune-modulating agents have been used in adjunct with steroids. Moreover, patients with evidence of heart failure or electrophysiological manifestations should be started on guideline-directed medical therapy. ICD therapy is also a vital consideration in the management of these patients. It can prevent complications of fibrotic conduction abnormalities and thus is recommended in patients with symptomatic advanced AV block or sustained VT. According to the Heart Rhythm Society Guidelines, ICD therapy is also recommended in asymptomatic CS patients with a high-risk profile, such as those with advanced AV block, extensive myocardial involvement, multiple episodes of VT, and severe left ventricular dysfunction [4,5,6].

## Conclusions

Advancements in cardiac imaging have helped with increased detection of CS, allowing for early treatment and prognosis. Even though corticosteroids have been the mainstay for therapy, there have been no definitive recommendations regarding the duration of therapy or appropriate dosing. Further, there have been no trials focused on the use of adjunctive immune-modulating therapies to allow for a shorter course of steroid use. It is vital to focus future research on the management of CS with adjunctive immunomodulators to prevent the long-term effects of corticosteroids. Lastly, conduction abnormalities are a common manifestation of CS and can increase the risk of sudden cardiac death. It is crucial for physicians to consider the use of ICD therapy in patients presenting with advanced AV block, bundle branch block, or VT. Adequate risk stratification can significantly reduce the incidence of sudden cardiac death in patients with CS.
